# The Twenty-Fourth Long Fox Memorial Lecture: Observations on Pain

**Published:** 1935

**Authors:** Macdonald Critchley

**Affiliations:** Neurologist, King's College Hospital; Physician to Out-patients, National Hospital, Queen Square


### The Bristol Medico-Chirurgical Journal

*"Scire est nescire, nisi id me*
*Scire alius sciret."*

WINTER, 1935.

### THE TWENTY-FOURTH LONG FOX MEMORIAL LECTURE:

DELIVERED IN THE UNIVERSITY OF BRISTOL ON TUESDAY, OCTOBER 22ND, 1935.

Dr. H. H. CARLETON, F.R.C.P., *in the Chair.*

BY

MACDONALD CRITCHLEY, M.D., F.R.C.P.,
*Neurologist, King's College Hospital ;*
*Physician to Out-patients, National Hospital, Queen Square.*

ON

### OBSERVATIONS ON PAIN.

---

WE have assembled to pay homage to the memory of Dr. Long Fox, whose birth 103 years ago was an important event in the medical history of Bristol. The lectureship associated with his name is an honour of which I am deeply conscious. I have endeavoured to select a subject which will not only be of general interest, but at the same time would have earned the

R

approval of Dr. Long Fox himself. I believe that the problem of pain, though an ambitious theme for the present lecturer, would fulfil these requirements. Some of the recent work would have had particular appeal to Dr. Long Fox. As a pioneer in the study of the sympathetic nervous system, he would have been interested in the modern discussion of whether pain sensations may be conducted by the autonomic system. As a neuro-psychiatrist, his views upon the psychological aspects of pain would have been especially valuable. It is upon this latter side that I particularly wish to dwell in this lecture.

INTROSPECTIVE ANALYSIS.

A study of the psychological processes which accompany and follow painful stimulation brings to light a number of interesting and important data.

What is popularly termed the "pain" of a pinprick or a burn may be analysed into three principal components. One may distinguish :—

1. A feeling of pressure (or piercing, cutting, heat, cold, etc.) which constitutes the *quality* of the pain.

2. A highly unpleasant sensation which constitutes the specific *pain-feeling*. This is of the same nature irrespective of the type of stimulus.

3. A feeling-tone, or emotion of strong displeasure.

It may be argued therefrom that pain (and pleasure) do not exist as isolated experiences, *i.e.* apart from sensation. This view, which is stoutly denied by some, is spoken of as the "quale theory" (Marshall) or as the "aspect theory" (Strong).

These three factors are present in most painful experiences, though very occasionally the first or the third may not occur. We may refer to the first component as "cognitive" and to the second and third as "affective" in nature. With mild pain cognitive factors are conspicuous and affective components slight; as the intensity of the stimulus increases, however, the cognitive factors tend to become swamped. Thus visceral pain is singularly devoid of qualifying sensation. Again, to quote Stout's illustration, when submitting to an intense pain, as when a tooth is extracted without an anæsthetic, the victim is conscious only of an overwhelming rush of pure pain, which so dominates attention as to crowd out the ability to recognize the other qualities of the sensation. James, indeed, states that in the case of intense acute pain it is impossible to recognize the nature of the stimulus, so that the pain of a cut would be indistinguishable from that of a burn or an electric shock. This is probably an over-statement.

Other psychological processes may accompany pain-sensations varying according to the nature, intensity and duration of the stimulus. Thus one can appreciate the temporal quality of the pain-sensation, whether intermittent or continuous; one can localize the sensation; one can compare or contrast the sensation with previous experiences. In particular other strong feeling-tones will appear in addition to the specific effect of displeasure. Thus there will arise an emotion of fear, panic, irritation, depression, anger, interest, etc., according to the attendant circumstances. The emotional colouring will differ considerably in the case of an accidental blow, from one received in anger, or from the ministrations of a surgeon, or from the effects of disease.

Depression is one of the commonest of the associated effects, and may be intense when the pain is particularly severe, protracted or recurrent, and especially when the prospects of relief are slight. It is striking, nevertheless, how comparatively rare is suicide on account of intractable suffering. Of 391 cases of suicide in Chicago during 1923 only 11 were attributed to a state of pain. It is interesting that 10 of these 11 cases occurred in males. In a series of 7,190 cases collected by Forbes Winslow pain is not once mentioned as a factor. Halbwach places it ninth in his list of the twelve principal causes of suicide. As Crichton Miller has pointed out, pain is probably a less frequent cause of suicide than other unpleasant sensations; more people take their life because of an incurable tinnitus than an intractable pain.

Very occasionally complex synæsthetic manifestations are associated with the feeling of pain. Thus the pain may evoke a conception of a visual character, or "photism," and the victim may describe his symptoms in terms of colour or of form. Perhaps the earliest example of this is to be read in Homer: "He removed the lid from the quiver and took out a fresh-winged arrow, harbinger of *black pains*." (*Iliad* IV.) Elsewhere* I have quoted many other examples of this synalgia, as it is called. There are three sets of circumstances which facilitate the appearance of this phenomenon. Thus it seems to be commonest (1) in blind subjects, (2) in states of intoxication particularly from mescal, and (3) in symbolist literature, where the language of one special sense is often made to serve for another.

Is there any limit of suffering beyond which no greater pain is felt however much the stimulus is augmented? We have no exact data here to help us, but the probability is that an acme of pain-feeling does exist. From the analogy of the conduction of pain

* *Brit. Med. Jour.*, 1934, ii. 891.

stimuli along the peripheral nerve this likelihood is enhanced. Whether this maximum lies within the powers of human endurance, or whether syncope, unconsciousness or collapse will occur before the upper limit is reached is open to question. In this connection one may refer to the suggestion of H. G. Wells that nociceptive stimuli if pushed beyond a certain point may transcend pain and actually become pleasurable.

PSYCHOGENIC PAINS.

Most of the research on the physiology and psychology has been made in cases where the pain sensation has followed a known and well-defined nociceptive stimulus or focus of disease. The problem of pain arising independently of any demonstrable stimulus is considerably more complex. In this group belong the various clinical types spoken of as hysterical, subjective, attentional, obsessional, or imperative, or, best of all, " psychogenic pain."

Pain is frequently described by victims of mental disease of many types. Various classifications of psychogenic pains have been offered, none of which is wholly satisfactory. Clinically we may distinguish pains which are generalized, vague and intermittent, from those which are strictly localized, well-defined and persistent. Or one may group the cases of psychogenic pain upon a basis of aetiology. Thus we may distinguish neurasthenic, hysterical, obsessional, and hypochondriacal varieties. It is noteworthy that severe pain is not a common symptom in cases of depression, unless hypochondriacal trends are conspicuous. This accords with the view that depression temporarily at least elevates the threshold for pain in normal individuals, and that physical and " moral " pain rarely coexist in any intensity.

Although psychogenic pain is usually diagnosed by eliminating physical disease, it is probable that there are certain clinical hallmarks which should from the start suggest a psychological origin. An inexplicable and precise periodicity (Bramwell), an excessive dependence upon the factor of suggestion, a relative intractability to drug treatment, together with a response to psychotherapy, are criteria which are often helpful. Still more important is a correct assessment of the factors operating in the patient's mind, and an intimate knowledge of the circumstances of the onset and the processes determining the nature and situation of the pain.

In states of neurasthenia, anxiety and reactive depression pains of a vague and diffuse character may be described. Rarely are they intense. Pressure headaches, backache, pains in the limbs, dyspepsia, precordial distress are characteristic symptoms.

As an excellent example of the type of headache characteristic of exhaustion states we may quote the following from Hugh Walpole's novel, *The Dark Forest* :—

"There was a peculiar little headache that I have felt nowhere else before or since, that attacked one on those early mornings. It was not a headache that afflicted one with definite physical pain. It was like a cold hand pressing upon the brow, a hand that touched the eyes, the nose, the mouth, then remained, a chill weight upon the head ; the blood seemed to stop in its course, one's heart beat feebly, and things were dim before one's eyes. One was stupid and chose one's words slowly, looking at people closely to see whether one really knew them, even unsure about oneself, one's history, one's future ; neither hungry, tired nor thirsty, neither sad nor joyful, neither excited nor dull, only with the cold hand upon one's brow, catching (with troubled breath) the beating of one's heart."

Pains occurring in hysteria and in obsessional states differ in being more severe and usually sharply delimited. The patient may complain of intense pain of a fairly complex type, restricted to the face, throat, head, hand or some other body segment, sometimes referred to as "topalgias." Gordon and Carleton have compared hysterical with thalamic pains, stating that both are affective in character, being poorly localized and of an intensity disproportionate to any exciting stimulus that may be detectable.

A variety of hysterical pain is that which regularly recurs at the same hour each day—the "habit pains" of Brissaud. Another rarer painful syndrome in hysteria is that described by Möbius as "akinesia algera." Here no suffering is felt while the patient is immobile: muscular activity precipitates severe pain, however. At first only gross effort is effectual, but finally the very slightest movement is accompanied by intense agony.

It is probable that the psychopathology of most cases of hysterical pain can be traced to a feeling of guilt, the pain serving as a means of expiation. This is well illustrated in the Hungarian tale, *The Bundle of Letters*, by Moritz Jokai:—

> A man consulted his doctor on account of an intense and circumscribed pain in the hand. This persisted despite cauterization and repeated free excisions. In despair the victim killed himself, leaving a confession to the effect that he had recently murdered his wife believing her to have been adulterous. Later, discovering that his wife had been innocent, he was overcome by remorse, and his hand, upon which a drop of his wife's blood had fallen, became the seat of an intractable psychogenic pain.

Usually it is difficult to understand why a particular region of the body should be affected by psychogenic pain. MacDougall has suggested that the symptoms become referred to that region which is in activity at the time of the psychic trauma. This is illustrated by his case of a girl with severe hysterical pains around the right shoulder: she had been told that her fiancé was unfaithful to her, and at the time of the shock had been reaching for something on a high shelf with the arm raised above the head.

Hypochondriacal pains are characteristically complex and bizarre. They belong to the class of cenesthopathic disorders, and may be localized or diffuse. In severe cases they recall some of the somatic delusions of the paranoid. The pains of hypochondria are very suggestive of a morbid lowering of the threshold of sensation, whereby somatic and visceral impulses, which normally do not reach consciousness, now become perceived and even interpreted as pain. This view is rather supported by the so-called "therapeutic test." As an index of psychogenesis in a doubtful case the response to a full dose of morphia may be noted. A pain of "organic" nature, dependent upon a peripheral stimulus, will be relieved, but not a pain of psychogenic character. In the case of a hypochondriacal pain some measure of relief is the rule; less, however, than would be expected in a case of a purely "organic" pain.

The same diagnostic difficulty is encountered in cases of persistent pain after an injury, which usually is either trivial in degree or remote in time. Such is the common experience in medico-legal practice. A workman sustains a minor injury and for years after complains of severe and disabling pain. The relative importance of the factors of structural disease, anxiety, sense of grievance, and desire for compensation may be most complex. Moreover, the greater one's knowledge of the problem the more difficult does it appear.

There exists a group of cases which are not readily assigned to a particular category, and the question of a possible psychogenesis is largely a matter of opinion. I refer to the so-called "psychalgias" including the coccyodynias, the post-herpetic neuralgias, and the pain of phantom-limbs after amputation. The following case, which I quote through the kindness of Sir Frederick Kay Menzies, illustrates the difficulties both of treatment and of diagnosis :—

J.O., a labourer, aged 57, was injured in January, 1913, by a cable-drum crushing the right foot. Though no bones were fractured the foot remained extremely painful, so that nine months later he had to be admitted to hospital. After three months' intensive physical treatment had failed to relieve him an amputation was performed just above the ankle-joint. Thereafter pain persisted not only in the stump but also in the phantom-foot. Two months afterwards a second amputation was made in the upper third of the thigh. Pain still persisted. A year later the flap was explored and the cut ends of the peripheral nerves freed and sectioned at a higher level. As this measure was ineffectual the great sciatic nerve was exposed and divided in two places. No relief followed. In 1922 a posterior rhizotomy was carried out on the lumbo-sacral plexus, and as this operation also did not help a chordotomy was made in the upper dorsal level of the cord, the antero-lateral tract being sectioned on the left side. Even this measure failed to relieve the symptoms. An alcoholic injection into the stump was the last therapeutic gesture. Seen twenty-two years after the original accident, the patient still complained of severe and persistent pain in the stump. The pain in the phantom-foot had spontaneously ceased a year or two previously.

In this case morphia would produce a slight and transient improvement.

Reference may also be made to those rare and interesting records of "sympathetic" pain, whereby the sufferings of one person are claimed to be actually shared by another. Although no scientific evidence exists to support this claim, the occurrence of sympathetic pains is widely accepted by the laity.

The conception of the victim's agonies being shared by a loved one or the pains of parturition by the young husband finds a place both in common speech and in literature.

We read that Perpetua—one of the early Christian martyrs—declared she felt the pain of the blows inflicted upon her father more severely than if she had received them herself. In recent times we read in a prison-letter from Sasha Berkman, anarchist and would-be assassin, " . . . the death of Russell especially affected me . . . he died a terrible death. The doctor charged the boy with shamming, but now he says it was spinal meningitis. . . . In some manner his agony seemed to communicate itself to me, and I began to experience the pains and symptoms that Russell described in his notes. I knew it was my sick fancy. I strove against it, but presently my legs showed signs of paralysis, and I suffered excruciating pain in the spinal column, just like Russell."*

At this point it becomes necessary to emphasize that the very existence of psychogenic pain has been challenged. Meyer Solomon in a detailed study of the problem has concluded that all pains are peripherally induced; that there are no subjective pains of primary central origin; that ideas, whether by suggestion or by fixation of attention or what not, cannot produce pains in the apparently normal, or in the neuropathic or psychopathic individual. The same opinion has more recently been supported by Walshe, who claims that there is no correspondence whatever between the pain described by the patient and the healthy and equable appearance and behaviour. As he says, the patient with psychogenic pain may continue for months as fat as a partridge, as rosy as an apple, and in a state of beatific calm.

These arguments, running directly counter to the views of most clinicians and psychologists, deserve some attention.

* Emma Goldman, *Living my Life*, vol. i., p. 292.

Obviously the conception of psychogenic pain is closely bound up with such questions as the memory and the mental imagery of pain. Everyone knows that in recalling a previous painful experience it is difficult, if not impossible, to conjure up an actual sensation of pain. One may readily evoke all the attendant emotions of displeasure and alarm, one may actually repeat the facial contortions and the gestures of the occasion, but it is doubtful whether anything further develops in the way of an actual perception. The idea holds equally true, of course, of special sense phenomena. How often can one evoke a true sensation of a visual, auditory, gustatory or olfactory nature, though one's imagery may be sharp and precise? The distinction is one, of course, between an image (or idea) and a percept (or sensation). Solomon agrees with Sidis that ". . . a sensation is not an intense idea, nor is an idea a weak sensation." According to Solomon, therefore, psychogenic pain is an imaginary pain, illusory in character. Had it actual existence in the sensorium it would be of hallucinatory nature.

Whether a rigid distinction exists between images and sensations is, however, open to argument, and belongs rather to a past era in psychology. We know from the experience of eidetic images and from the photisms of synæsthetic phenomena that intermediate experiences may occur belonging neither to the category of ideas nor of percepts. Mental imagery is extremely variable in intensity and clearness, and in some persons may be of almost perceptual vividness. In neuropathic and psychopathic subjects, just as in certain asthenic and toxi-infective states, it is highly probable that an intensification of imagery may occur, especially along particular aspects.

A recent study of tactile images by Bromberg and

Schilder has demonstrated how variable is this process among normals, and how keenly the faculty may be developed in some cases. Ribot has made a particular note of the acuity of pain imagery and recollection based upon a series of investigations in normal subjects. Almost always there was a keen reproduction of all the motor and mimic elements of suffering. Although in many cases the actual painful element could scarcely be revived, if at all, in many other instances it would reappear with the utmost clearness. Fouillée, for example, stated that one could reproduce in consciousness, even though incompletely, the sensational quality of, for example, a toothache. To quote his own words :—

> "To this end we must employ an indirect method. This procedure consists in directly calling up the images and motor reactions which accompany or follow toothache. I make the experiment, localizing my thoughts in one of the molars of the right side ; then I wait. The first thing to revive is a vague and general state, common to all painful sensations. Then this reaction grows more precise, as I concentrate my attention more and more on the tooth. At last I feel a greater influx of blood into the gum, and even throbbings. Then I represent to myself a certain movement passing from one point of the tooth or gum to another ; this is the passage of the pain. Thus I also revive the motor reaction carried by the pain, the convulsion of the jaw, etc. Finally, by thinking fixedly of all these circumstances, I end by feeling in a more or less dull way the rudiments of shooting pains. In an experiment just made I have brought on real toothache in a molar."

It is also affirmed by those who deny the existence of psychogenic pain, that pains cannot be felt in another set of purely ideational circumstances, namely in dreams. Common experience teaches, of course, that the pain of a toothache or boil may assert itself during sleep, either by colouring the dreams with a typical painful quality, or by the creation of a specific structure of symbolisation, or by a peculiar compulsive type of dream, as Marcel Proust has described :—

"When we have gone to sleep with a maddening toothache and are conscious of it only as a little girl whom we attempt, time after time, to pull out of the water, or as a line of Molière which we repeat incessantly to ourselves, it is a great relief to wake up, so that we can disentangle the idea of toothache from any artificial semblance of heroism or rhythmic cadence."*

In the same way a sudden painful stimulus applied to a sleeping person may weave itself rationally into a dream-pattern, and often, oddly enough, in a retrospective manner. Thus a complicated dream-story may culminate logically in a climax of pain which exactly coincides with the acute painful stimulus.

Pain may appear in dreams under another set of conditions. Dr. Bernard Hart has described to me a patient whose tooth had been painlessly extracted under hypnosis. During sleep the same night he awoke with a sudden pain in the socket. None of these types of dream throw light upon the question of psychogenic pains, however, as these sensations are obviously peripherally induced. The crucial answer would be found in the occurrence of a sudden dream-pain, arising quite independently of any stimulus sufficiently severe to arouse the sleeper. It is commonly denied that such pains occur in dreams, but the grounds for such disbelief are not altogether convincing. Certainly during the enacting of a dream-story one seems to see, hear, feel and taste with perfect naturalness, and I am inclined to believe to suffer pain also. Were it otherwise the dream-story would be devoid of all verisimilitude and never succeed in

* *Swann's Way,* vol. i.

deceiving the dreamer. The memory of pain is notoriously short, and it is not surprising that on waking little or no recollection of the pain-sensations should remain.

An important argument in favour of the existence of psychogenic pains lies in the fact that pains can be engendered by hypnotic suggestion. Of this observation there can be no denial. Meyer Solomon protests, however, that hypnotically-induced hallucinations, including pain, are not really experienced but are only delusions. The question, therefore, resolves itself into a distinction between pains which are really felt and those which the victim only imagines he feels. "As if pain, *qua* pain, had any existence except as a subjective experience. If a pain were imaginary it would cease to be a pain." (MacCurdy.) The distinction is obviously one outside the sphere of common sense.

To argue that psychogenic pains are really only some other form of unpleasantness is manifestly false. Although the term "pain" is often loosely and inaccurately applied by many neurotics to some of their abnormal subjective symptoms, true "hysterical" pains are as real to the victim as somatic pains which have previously been experienced. No difference either in severity or in quality can be traced by even the most intelligent and introspective of patients.

The argument as to the absence of physical signs of pain in psychogenic cases scarcely applies. On the one hand we believe that there are no unequivocal marks which accompany pains of any type, and we are all familiar with patients with severe neuralgia who sleep and eat well and do not lose weight. Conversely, all the traditional concomitants of pain may be found in psycho-neurotics; the victim of psychogenic pain may display every variety of the *facies dolorosa* recorded in Killian's atlas. Those who accept physical signs as characteristic of pain may recall that even with psychogenic pain objective manifestations have been recorded. Thus pupillary dilatation, tachycardia and a slight degree of vaso-constriction have been noted during the mere recollection of painful experiences. Especially important are the researches of Levine, who discovered a very definite psychogalvanic response in cases of hysterical pain and also in cases of pain suggested during an hypnotic state.

### SENSITIVITY TO PAIN.

It is common knowledge that individuals vary considerably in their susceptibility to pain. An identical stimulus applied to a series of normals would evoke descriptions of a widely-divergent character. Allowance must be made, of course, for the factor of language, for a source of error may be introduced in the verbal descriptions.

Surprisingly little research has been carried out upon this aspect of the pain-problem. Macdonald is one of the few to carry out algometric experiments on a large scale. His conclusions confirm the general impression, namely that women are more sensitive to pain than men, young subjects than old, and that members of the educated, well-to-do and leisured classes are more susceptible than the illiterate, poorer, labouring members of the community. Urban dwellers are more sensitive than rural. Libman has classified individuals into "sensitives" and "hypo-sensitives" according to their reaction to a standard pressure-pain stimulus. His impression was that hyposensitives generally feel less pain in the course of disease and tend to localize imperfectly their painful sensations. He declares that the usual text-book descriptions of symptomatology apply only to sensitives, and that diagnostic difficulty is apt to arise in the case of disease in hyposensitives. Libman's opinion in this way differs from the earlier observations of Griffing, who found that sensibility to one form of stimulation does not necessarily serve as an index to pain sensitivity in general.

Racial factors are, of course, highly important. Thus white races are much more susceptible to pain than are the uncivilized. The only possible exception to this generalization is the claimed sensitivity of some of the Melanesians (Malinowski). Libman found that the proportions of sensitives was unusually high amongst Jews and low amongst the Red Indians. A later investigation by Macdonald upon a series of United States Congressmen also suggested that race was a significant factor in pain-sensitivity. He found that those of Dutch, Scotch and English descent were the least sensitive to pain (in that order), while those of the Irish, French and Welsh lineage were most sensitive.

Undoubtedly the factor of acquired hardihood is all-important. Races and individuals who have been sheltered from hardships and pain stimuli display susceptibility to pain foreign to those whose upbringing has been punctuated by suffering. Of a series of ninety-seven New York pugilists only six could be classed by Libman as belonging to the group of sensitives. This observation may also explain the "toughness" as well as the brutality of so many of the criminal population.

It is well known also that an individual's sensitivity to pain may vary according to a number of intrinsic and extrinsic factors. We are reminded that under the stress of emotion—excitement, anger, pleasure—the threshold for pain becomes temporarily raised. The footballer may be unconscious of the injury which raises a bruise; the soldier may be unaware he is hit until he sees blood flowing from his wound. Oddly enough, the threshold for other cutaneous sensations is not necessarily raised at the same time, and the soldier's attention may be directed to his wound not by the pain but by the warmth or dampness of the blood-flow. At other times pain-sensitivity may be temporarily lowered. Introspection, self-analysis and close attention, for example, will augment sensation. Cold and hunger may also depress the threshold. Fatigue probably acts in the same way, though this view is not confirmed by the observations of Patrick and Gilbert, who found no change in the maximal and minimal thresholds for pain in a subject kept awake for ninety hours. Loss of weight and debility usually render the subject more sensitive to painful impressions. Mild depression, particularly when accompanied by agitation, increases pain susceptibility, but deeper states of depression bring about a certain measure of indifference towards nociceptive stimuli.

Amongst emotions of this type may be included the fear of death. This overwhelming apprehension may drive the victim to submit lightly to all manner of painful manipulations. Dr. Johnson, who was always afflicted with a great dread of death, declared during his last illness that he would give one of his limbs for another year of life. As the surgeon was making punctures in his legs to relieve œdema Dr. Johnson cried: "Deeper! Deeper! I want length of life, and you are afraid of giving me pain, which I do not value."

One or two factors may influence pain-sensitivity either in a positive or a negative direction. Thus expectation of pain may serve to augment the effect of the stimulus when it arrives, or—in some cases—to reduce it. Similarly the factor of an already existing painful state may serve to enhance or to inhibit the effect of a fresh pain stimulus. Hippocrates' dictum : " Of simultaneous pain in two places, the lesser is obliterated by the greater, " is only partially true.

### ANALGESIA AND ALGOPHILIA.

We may now consider those interesting cases where pain sensations are deliberately sought and endured, and in which some relative elevation of the threshold for pain seems to exist. In some instances there appears to be a complete analgesia, so that noxious stimuli can be borne with indifference. In other cases a certain pleasure seems to be associated with the endurance of suffering (algophilia). Both types of case will be considered together, for it is probable that the appreciation of pain is markedly reduced in the latter group also.

It is common to find oneself fingering a painful scar or probing an aching tooth with the tongue : in this way a dull ache is converted into a sharper type of pain, and curiosity as to the progress of the original lesion is satisfied. This type of stimulation is never applied to a severe or prolonged painful sensation, however. Some types of " worrying " chronic pain are relieved by the distraction of an added acute pain. Thus the dull ache of a neuritis may be relieved by the keener sensation of a blister or a puncture. Sylvia Pankhurst relates that her father, during his last illness, used to plunge his hands into the sharp thorns of the hedgerows in order to assuage his pain.

Still commoner is it to find that pain sensations are sought in order to swamp the discomfort of an intolerable dysæsthesia, and the skin may be scratched and torn in the effort to relieve an itching.

More remarkable is the fact that in some psychological disorders a complete corporal apathy or cenesthesia may reach the pitch of being unendurable. This phenomenon may occur in drug addiction, and some believe that the actual piqures are pleasurable to the morphinist, quite apart from the associated dosage. This type of algophilia is well expressed in the autobiography of Brenda Dean Paul :—

"I had mentally lost my sense of taste and smell, like a spiritual cold, that left life savourless and empty. I could bear the ennui no longer, so one night had found temporary relief in piercing my wrist with the jagged piece of tooth-glass, time after time. . . . Dr. Viney had asked Lord Dawson of Penn and Dr. Harold Chappell (the gynæcologist) to come round, and I remember their hearty congratulations on my stoicism when they put four stitches into my wrist, little knowing that each stab of the needle came as a welcome relief to me in my deadly apathy."*

Some of the grossest examples of the auto-infliction of pain with apparent insensitivity are to be encountered among the insane and certain mental defectives. Idiots and imbeciles often cause self-injury by biting or pummelling themselves, or beating their heads against the wall. Psychotics have been known to burn or scald themselves severely, chew broken glass, and perform all manner of terrible auto-mutilations. Goodhart describes a girl who avulsed her eyeballs. Conn's patient fractured her phalanges, dislocated her thumbs, and tore off her ear. Weatherley has described the case of a woman who had made many attempts upon her life. On one occasion she was discovered in flames : she had torn out her eyes and had placed flaming hot coals in her vagina, anus and armpits. At the same time she was roaring with laughter and cried exultingly : "Now I've got you, you devils ! "

* Brenda Dean Paul, *My First Life*, p. 178.

The explanation of this is problematical, and may indeed vary from one case to another. In defectives the pain sensation may perhaps be incorrectly interpreted. Perhaps in the majority of cases the self-infliction of pain represents an effort to appease a sense of guilt or to atone for a misdeed, real or imaginary. There is in this way a close and interesting association between the self-infliction of pain in the insane and the subjective pains of the hysteric.

A temporary rise in the threshold for pain has already been mentioned in association with depressive states. When the frenzy of despair drives the victim to the point of suicide, it is probable that pain-sensitivity is still further inhibited. Suicides do not necessarily seek the most painless or rapid methods, and sometimes a very elaborate, protracted and agonizing mode of exitus is chosen and persevered with. Thus the victim may slash his forearms with a razor twenty or thirty times in the effort to sever an artery, and finally cut his throat by a series of incisions. An examination of a number of would-be suicides has convinced me that a state of relative analgesia had been present during the attempt.

We are familiar with widespread and profound reductions in pain-sensitivity in cases of hysteria. Here consciousness may or may not be impaired. In the latter case there is a strong similarity to the trance-like states of a spiritualistic medium, in whom complete analgesia can be demonstrated. Hysterical states with intact consciousness are comparable with other conditions in which an overwhelming surge of emotion dominates consciousness. One recalls the ecstatic states of the martyrs and devotees of all religions.

The Moslem dervishes, for example, engage in a prolonged ritual, and so produce a state of divine frenzy in which they cut themselves, and eat snakes, hot coals and glass. The orgies of self-torture practised by the Aïssaouas are preceded by elaborate rituals in which chants and rhythmical movements of the body are conspicuous. The priests of Baal used to indulge in a rain-making ceremony, wherein they would gash themselves with knives until blood gushed out. In the Middle Ages bands of flagellants went from city to city singing psalms and scourging themselves.

Some idea of the ecstatic state engendered by these practices can be gleaned from the personal descriptions of St. Theresa, the flagellant: "In this agony of suffering there is such a great happiness that I cannot describe it. It is an unspeakable martyrdom and of delight together. . . . I saw a long golden lance in the angel's hands pointed with fire. Now and again he would plunge it through my head and bury it in my entrails: in withdrawing it he seemed to drag them out with his lance and leave me completely filled with God's love. The pain of that wound was so intense that it tore from me feeble sighs which I could scarcely breathe; but that indescribable martyrdom allowed me at the same time to taste the gentlest delights, so that I did not want it to come to an end, nor to find happiness outside my God."

Again, under the torture of scourging Maria-Magdalena of Florence is said to have exclaimed: "Enough! Do not increase this flame which is devouring me; I do not want this kind of death—it is too pleasant and too exquisite!"

Throughout the centuries martyrs have displayed during their torture a fortitude and even exultation which can only be attributable to an ecstatic state which has profoundly modified the threshold of pain. All the evidence points to a highly abnormal psychological state while under ordeal. We read that St. Lawrence joked while being roasted on the grill, and facetiously requested the executioners to turn him over as he was cooked on the one side. Gibbon narrates that the early Roman martyrs used to discover a sensation of joy and pleasure in the midst of the most exquisite tortures: they would deliberately provoke the lions to which they had been thrown, exasperate the executioners, and cheerfully leap into the flames which were being kindled for them. The ancient philosophers, we are told, used to regard their behaviour more with astonishment than with admiration. During the persecution of the heretic Donatists, also, the same frenzied suicides and desire for martyrdom were observed.

An interesting and significant point is mentioned by Foxe in his Martyrology. We read that when Nichomachus was put to the rack he bore the torture with patience and great resolution ; when on the point of death, however, he abjured his faith. Immediately he fell into the greatest agonies, collapsed and died. We read also that Zoë, the wife of one of the gaolers, suddenly became a convert, and at the same time a loss of speech immediately became restored.

The cult of pain is also followed by ascetics of all types. Here the basic motive is probably one of discipline, the development of the spiritual life by the denial or mortification of the flesh. To the Stoic philosopher pains were an evil over which it was possible to triumph by a proper exercise of discipline and control. Perhaps the highest form of asceticism is to be found among the Paramahamsas of India, who are "equally indifferent to pleasure and to pain, insensible of heat or cold, and incapable of satiety or want."

Ascetics have been found among all religions and in all times. Christianity can point to St. Simeon Stylites, who lived for thirty years upon a column seventy-two feet high and four feet square at the top ; and also to St. Francis of Assisi and to St. Anthony.

MacDougall has attempted to explain — not altogether convincingly — the fortitude of such ascetics as the deliberate suppression of the wish to escape to such an extent that the painful sensations cease to be painful. To quote his own words : "The training and belief of such persons render them capable of voluntarily submitting to the torture and of suppressing by strong volition the tendency to struggle to escape evoked by the strong sense-impressions ; and in so far as they succeed in this the experience ceases to be painful." Others would ascribe the sacrifices of the ascetic merely to a wish to find favour with the deity. In this way the pain sensations are sublimated into a feeling of spiritual satisfaction.

A minor variant of this philosophy is to be found in communities where submission to pain is practised as a means of attaining hardihood, virility and power. Herein lies much of the creed of the Spartans and of the Samurai of Japan. The same idea exists amongst many primitive races. One of the motives for tattooing is the factor of pride and conceit in the ability to bear pain as denoting not only a certain social status but also a visible mark of virility. For the same reason the Fulami youths submit boastfully to ordeals by flagellation.

There are, of course, other and less exalted reasons for self-injury and automutilation amongst aboriginal races. It is widely believed that disease can be averted from a bodily organ by sacrificing or marking some part of it. Thus we find among the Malays filing of the teeth to prevent decay, extraction of teeth to prevent the mouth closing up (the Dieri), piercing the nasal septum to avoid gummatous ulceration (the Cadiacks), and the insertion of plugs or sticks through the ears, nose and lips to divert evil. Probably one of the reasons for circumcision was to avoid disease, or—what is more likely—to ensure the well-being of the whole by the sacrifice of a part. Still another primitive belief is that by inflicting oneself with pain one can propitiate the demons which produce pain and disease.

The Moslem fakirs and the Indian sadhus belong to a somewhat different category. Numbering between three and five millions, they travel around the country performing numerous feats of mutilation. They will eat glass, lie on a bed of spikes, hang from hooks, roast themselves over a slow fire, walk over glowing coals, pass skewers through their cheeks, tongue or neck. They can scarcely be compared with the frenzied participants of a religious orgy, like the dancing dervishes or flagellants. Nor do they strictly belong to the company of pious ascetics. On the contrary, many of their activities are carried out deliberately and in cold blood in the hope of alms, the performance bearing all the marks of showmanship. For this reason their feats become all the more interesting and difficult to explain.

Until recently no attempt has ever been made to study scientifically their feats of endurance. Lately, however, J. H. Hunt has described with a number of brilliant photographs a series of visits which he and his father paid to a family of Rafaee fakirs in Hyderabad. They were permitted to examine the performers closely before, during and after their activities, as well as to make cinematographic records. The feats include the passage of skewers through the abdominal wall, the cheeks, tongue and neck, and the levering forward of the eyeball out of the socket. Hunt concluded that the fakirs at times feel considerable discomfort, especially the younger performers and the novices. He believes that originally the practices are extremely painful, but frequent repetitions not only serve to raise the threshold for pain, but also tend to lay down a considerable amount of scar tissue which is, of course, insensitive. Moreover, one must add the factors of religious excitement which attend some of these exhibitions, and secondly the different scale of values permitting the fakir to endure terrific amounts of physical suffering in order to attain a future bliss. Hunt concludes that there are no mysterious physical qualities attached to these fakirs. "It is in their minds they are different. The qualities of patience, perseverance and acceptance of suffering are developed to a degree quite pathological."

In view of the recent demonstration at Carshalton, a few remarks upon the classic "fire-walk" may be of interest. This ritual has been practised for at least 3,000 years in almost every part of the globe. Nowadays it is most often associated with India and Polynesia. Two varieties occur: the "stone-walk," where the performer walks through a furnace over a layer of flat stones, and the "ember-walk," where the fire is topped with smouldering wood and sticks. Though many descriptions and attempts at explanation have been made, the most comprehensive study is that of E. S. Thomas. This author believes that there is nothing in the fire-walk transcending physical or physiological laws. He regards as significant the quick formation of an insulating layer of ash (in the ember-walk), the low-conductivity of the rock (in the stone-walk), the effects of exaltation and self-suggestion, strengthened by ascetic training, in reducing the temperature and increasing the insensibility of the body, aided by cold bathing, and lastly the calloused foot-soles of the walkers and their quick, short steps. Hunt also includes the possible existence of a "spheroidal state," whereby the feet are protected against the heat by a thin layer of water vapour.

Other apparent examples of invulnerability to fire may be mentioned. A well-known circus attraction is found in the so-called "human salamanders," who will permit flames or hot coals to enter the mouth with impunity. Sometimes the hands and arms share the thermanalgesic properties. Not only are evidences of pain absent in such cases, but no marks of burning or singeing are visible. One of the finest descriptions of this type of side-show is contained in Evelyn's diary, as pointed out by Thurston: "October 8th, 1672.—Richardson, the famous fire-eater—devoured brimstone and glowing coals before us, chewing and swallowing them; he melted a beare-glasse and eate it quite up; then taking a live coale on his tongue, he put it in a raw oyster, the coal was blown on with bellows till it flamed and sparkled in his mouth, and so remained until the oyster gaped and was quite boiled; then he melted pitch and wax with sulphur, which he drank down as it flamed; I saw it flaming in his mouth a good while; he also took up a thick piece of yron, such as laundresses use to put in their smoothing-boxes, when it was fiery hot, held it between his teeth, then in his hand, and threw it about like a stone, but this I observed he cared not to hold very long; then he stood on a small pot, and bending his body, took a glowing yron with his mouth from between his feete, without touching the pot or ground with his hands; with divers other prodigious features." Thurston has classified the cases of human incombustibility into four categories: (1) those of a "miraculous" nature, often associated with martyrdom, as in the case of St. Polycarp ; (2) where some perversion of the laws of physiology seems to be concerned ; (3) spiritualistic mediums ; and (4) the "fire-walkers." Where precisely belong the "fire-eaters" of the side-show is uncertain. Undoubtedly some of the performances are partly, if not wholly, of the nature of a conjuring trick, depending to some extent upon the phenomenon of the spheroidal state. At times, also, some preparation of the skin takes place. There is also a possibility that some of the performers are capitalizing a well-known morbid analgesia, as best exemplified by syringomyelia.

Perhaps the best known examples of algophilia are found in the phenomena of masochism, where sexual gratification is afforded by physical suffering or mental humiliation. There is an abundant literature upon this subject, both psychological and belletristic, and nothing is to be gained by repetition. One may merely echo the opinions of Krafft-Ebing, as offering the most convincing explanation, that the pain in these circumstances is merely overwhelmed by an associated pleasure to such a degree that in retrospect the pain becomes regarded as the symbol of pleasure.

Lastly, one may refer to those extremely rare instances of "natural analgesia." Here there seems to be a question of a physiological elevation of the threshold for nociceptive stimuli to such heights that pain seldom, if ever, enters consciousness. In an earlier paper I have given details of several such cases, where wounds, surgical operations, fractures and disease occurred in the complete absence of suffering in otherwise normal subjects.

Natural analgesia must not be regarded as an altogether desirable phenomenon, however. Langdon Brown has aptly quoted the fairy-tale of the woodcutter who was granted his wish of becoming insensitive to pain ; very soon he succumbed to the effects of burns and other injuries. The biological function of pain is usually regarded as one of protection; pain betrays the presence of injurious influences or of disease and stimulates the organism to flight. It matters little that in its rôle of "vigilant sentinel" its action is frequently crude, inadequate and imperfect. A race of beings devoid of pain-sense would possess but slender chance of survival. The memory of pain, although short, is all-important in conditioning behaviour. As Richet said, pain may well be regarded as one of the bases of intelligence, since the memory of pain determines the conduct of all beings above the rank of pure automata.

It does not follow that the effects of pain are beneficent, as some have claimed. Under the arresting title of "Pain is an Incentive," St. John Ervine, in a recent paper has attempted to show that suffering has stimulating powers of immense therapeutic value; that it may constitute a positive virtue and in some persons act as a spur to work. Such doctrines are both antiquated and mischievous.

We are reminded of some of the superstitions of the last century, in which unfortunately medical men were gravely involved. The controversies aroused by the introduction of anæsthesia into surgery and obstetrics form a regrettable chapter in medical history. Less than ninety years ago we find a surgeon proclaiming that "pain was a wise provision of nature, and patients ought to suffer pain while their surgeons were operating; they were all the better for it and recovered better." A protracted and acrimonious warfare ensued, with Biblical quotations and abuse as the chief instruments of offence. The cause of the humanitarians was eventually saved by the happy recollection of an illustration from Genesis, wherein the Deity, wishing to fashion a woman from Adam's rib, first threw Adam into a deep slumber. This text turned the enemies' flank, but their crushing defeat came from an unexpected quarter. A royal event was anticipated at Windsor, and the Queen gave an example to the nation and quietly submitted to chloroform during her confinement with

Prince Albert Edward. Anæsthetics thenceforward were accepted as respectable.

To-day we distinguish between pain as an index of incipient disease and pains which persist after their function as a monitor has been served. We tend to subscribe to the humaner philosophy voiced by Sir James Young Simpson, the pioneer of chloroform anæsthesia ". . . all pain is *per se*, and especially when in excess, destructive, and even ultimately fatal in its action and effects. It exhausts the principle of life. It exhausts both the system and the part. Mere pain *can* destroy life."

### BIBLIOGRAPHY.

Marshall, H. R., *Pain, Pleasure and Æsthetics*. 1894.
Strong, C. A., *Psychol. Record*, 1895, ii. 164, 329.
Gordon, R. G., and Carleton, H. H., *Brain*, 1923, xlvi. 221.
MacDougall, *Outline of Abnormal Psychology*.
Solomon, M., *Med. Record*, 1917, xcii. 314.
Walshe, F. M. R., *Brit. Med. Jour.*, 1930, i., 18th January.
Ribot, *Psychology of the Emotions*.
Fouillée (quoted by Ribot).
Levine, M., *Johns Hopkins Hosp. Bull.*, 1930, 331.
Killian, *Facies Dolorosa*.
MacDonald, A., *Boston Med. and Surg. Jour.*, 1899, clv. 146.
Libman, E., *Trans. Assoc. Amer. Phys.*, 1926, xli. 305.
Griffing, H., *Psychol. Review*, 1896, iii. 412.
Malinowski, *The Sexual Life of Savages in North-Western Melanesia*.
MacDonald, A., *Congressional Record*, 1932, 72nd Congress, 1st Session.
Patrick, G. T. W., and Gilbert, J. A., *Psychol. Review*, 1896, iii. 469.
Gibbon, E., *Decline and Fall of the Roman Empire*, vol. ii., chap. xvi.
Foxe, *Book of Martyrs*, Book i., Section ii.
MacDougall, *Social Psychology*.
Hunt, J. H., *St. Bart's Hosp. Jour.*, 1934, xlii. 11, 31, 55.
Thomas, E. S., *Proc. Soc. Psychical Res.*, 1934, xlii. 292.
Thurston, H., *The Month*, February-March, 1932, 144.
Richet, *Trans. 3rd International Congr. Psychol.*, 1896.
Ervine, St. J., *Good Housekeeping*, 1935 (September), 32.

